# Giardiavirus Internal Ribosome Entry Site Has an Apparently Unique Mechanism of Initiating Translation

**DOI:** 10.1371/journal.pone.0007435

**Published:** 2009-10-14

**Authors:** Srinivas Garlapati, Ching C. Wang

**Affiliations:** Department of Pharmaceutical Chemistry, University of California San Francisco, San Francisco, California, United States of America; Institute of Protein Research, Russian Academy of Sciences, Russian Federation

## Abstract

Giardiavirus (GLV) utilizes an internal ribosome entry site (IRES) for translation initiation in the early branching eukaryote *Giardia lamblia*. Unlike most of the viral IRESs among higher eukaryotes, which localize primarily within the 5′-untranslated region (UTR), the GLV IRES comprises 253 nts of 5′UTR and the initial 264 nts in the open-reading-frame (ORF). To test if GLV IRES also functions in higher eukaryotic systems, we examined it in rabbit reticulocyte lysate (RRL) and found that it functions much less efficiently than the IRES from the Encephalomyocarditis virus (EMCV) or Cricket paralysis virus (CrPV). In contrast, both EMCV-IRES and CrPV-IRESs were inactive in transfected *Giardia* cells. Structure-function analysis indicated that only the stem-loop U5 from the 5′UTR and the stem-loop I plus the downstream box (Dbox) from the ORF of GLV IRES are required for limited IRES function in RRL. Edeine, a translation initiation inhibitor, did not significantly affect the function of GLV IRES in either RRL or *Giardia*, indicating that a pre-initiation complex is not required for GLV IRES–mediated translation initiation. However, the small ribosomal subunit purified from *Giardia* did not bind to GLV IRES, indicating that additional protein factors may be necessary. A member of the helicase family IBP1 and two known viral IRES binding proteins La autoantigen and SRp20 have been identified in *Giardia* that bind to GLV IRES *in vitro*. These three proteins could be involved in facilitating small ribosome recruitment for initiating translation.

## Introduction

Internal ribosome entry site (IRES) mediated translation is an alternative mechanism of translation initiation adopted by many viruses and some cellular mRNAs among higher eukaryotes [1]–[3]. Among the members of *Picornaviridiae* and *Flaviviridiae* families, the IRESs are primarily located in the 5′untranslated regions (UTRs) of the transcripts [3]. However, some rare exceptions such as the Dicistroviruses with IRES elements in the intergenic regions (IGR) [4], [5] and HIV 2 having an IRES element entirely in the downstream coding region have been identified [6]. For the cellular mRNAs, IRESs are primarily located in the 5′ UTRs and often function in a cell-cycle-dependent manner [7], [8].

In the cap-dependent translation initiation, the 40S ribosomal subunit complexed with initiation factors eIF3 and eIF2-GTP-Met.tRNA_i_ (43S pre-initiation complex), binds to the 5′ cap structure of the mRNA via the eIF4F complex and subsequently scans for the start codon [9]. In contrast, the IRES mediated translation initiation involves direct recruitment of the translation machinery that positions the 40S small ribosomal subunit onto the start codon [1]. The process of 40S ribosome binding to IRES varies with different types of IRES and also with the protein factors involved [10], [11]. For example, the poliovirus (PV) IRES and Encephalomyocarditis virus (EMCV) IRES require all the canonical initiation factors except for the cap binding protein eIF4E to recruit the 43S pre-initiation complex [12]–[14], whereas the Hepatitis C virus (HCV) IRES requires only the binding of initiation factor eIF3 for efficient recruitment of naked 40S ribosome [15]–[17]. In contrast, the IRESes present in the intergenic regions (IGR) of Dicistroviruses do not require any initiation factors for binding to the 40S ribosome [18]–[20].

In addition to the initiation factors, some of the viral IRESs also bind non-canonical protein factors known as IRES *trans*-acting factors (ITAFs) that have been shown to stimulate IRES activity. For instance, La autoantigen was shown to enhance the IRES activity of PV IRES [21], [22], HCV IRES [23]–[25], EMCV IRES [26] and Coxsackievirus B3 IRES [27], whereas the polypyrimidine tract binding protein (PTB) was found to bind and induce conformational changes in EMCV IRES [28]–[30], PV IRES [31], and Foot-and-mouth disease virus (FMDV) IRES [30]. Recently, a splicing factor SRp20 was also implicated in PV IRES activity via an interaction with Poly (rC) binding protein 2 (PCBP2) [32]. A distinct class of RNA binding proteins containing three K-homologous (KH) domains such as heterogeneous nuclear ribonuclear proteins hnRNPE1 and hnRNPE2 have also been shown to bind to PV IRES [33], [34] and Hepatitis A virus IRES [35]. For many cellular IRESes, several proteins that are involved in mRNA splicing and transport were identified as *trans*-acting factors [2], [3], [36], [37].

**Figure 1:**
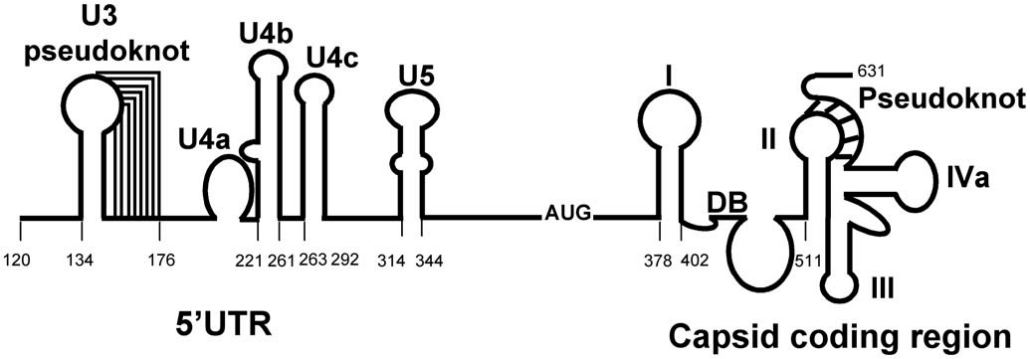
The essential secondary structures of GLV IRES. Secondary structures identified in the GLV IRES by chemical/enzymatic structure probing and site-directed mutagenesis.

Viruses of the *Totiviridiae* family represent a small group of double stranded RNA viruses that infect protozoan parasites and lower fungi [38]. Giardiavirus (GLV), a member of the *Totiviridiae* family, inhabits the cytoplasm of an early branching protozoan parasite *Giardia lamblia* [39]. Its transcript encodes two proteins: a major capsid protein of 100 kDa and a minor 190 kDa gag-pol fusion protein produced via a –1 ribosomal frame-shift [40], [41]. It lacks a 5′ cap structure but contains a highly structured 5′ untranslated region (UTR) [42]. The 5′UTR alone is not sufficient to initiate the translation of the viral transcript. It needs to combine with a 264 base stretch of the downstream coding sequence to function as an IRES in *Giardia* [43], [44]. This unusual IRES has been subjected to a thorough structure-function analysis that identified several complex secondary structures essential for IRES function (Fig. 1) [45]–[47]. However, how GLV IRES recruits the host translation machinery to initiate protein synthesis in *Giardia* is poorly understood.

The viral host *Giardia* is known to possess the translation machinery with many unusual features, such as the involvement of a 70S instead of a 80S ribosome [48] and the translation initiation factors that are either missing or structurally divergent as compared to that of the higher eukaryotes [49]. To learn whether the GLV IRES may function in a novel mechanism of translation initiation due to the unusual translation machinery in *Giardia*, we tested its function in rabbit reticulocyte lysates (RRL) and found significantly reduced IRES activity with very simple structural requirement. Further analysis indicated that GLV IRES may not require a pre-initiation complex for initiating translation both in *Giardia* and in RRL, nor does it bind to *Giardia* small ribosomal subunit *in vitro*. A member of the helicase family GlIBP1 is identified from the affinity purified IRES-protein complexes formed in *Giardia* lysate. In addition, two ITAF homologues La autoantigen and SRp20 are identified in *Giardia* that exhibited binding to the GLV IRES *in vitro*. These results suggest a mechanism of GLV IRES binding to the ITAF protein factors prior to ribosomal recruitment.

## Results

### The function of GLV IRES in RRL

**Figure 2:**
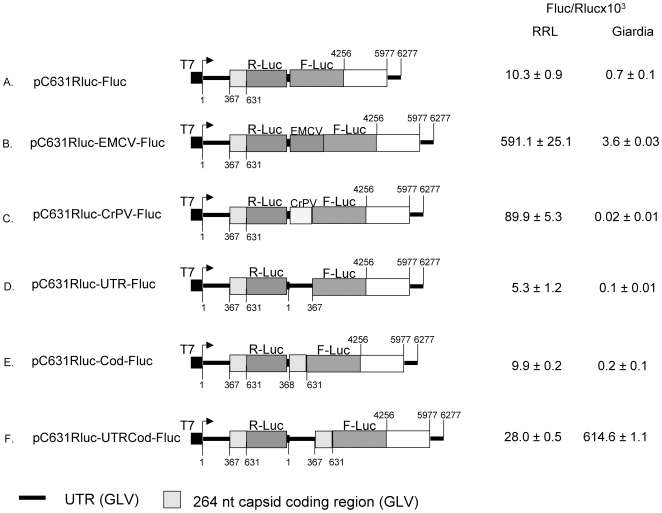
GLV IRES activity in RRL and in transfected *Giardia* WB trophozoites. Transcripts were synthesized from various dicistronic cDNA constructs each consisting of the Renilla luciferase Rluc (shaded box) and Photinus luciferase Fluc (hatched box) genes flanked by the 5′ and 3′ portions of GLV cDNA (the coding region, dash-hatched box; 5′ and 3′ UTRs, black bars) and located downstream from the T7 promoter (black box). The Rluc and Fluc cistrons are separated by 10 nucleotides in the control pC631Rluc-Fluc transcript (indicated by small black box). Schematic diagrams of dicistronic cDNA constructs (1) pC631Rluc-Fluc, (2) pC631Rluc-UTR-Fluc, (3) pC631Rluc-Cod-Fluc, (4) pC631Rluc-UTRCod-Fluc, (5) pC631Rluc-EMCV-Fluc, and (6) pC631Rluc-CrPV-Fluc are presented. Reaction products from RRL and transfected *Giardia* trophozoites were assayed for Ruc and Fluc activities and the IRES activity observed with each transcript was expressed as a ratio between the two luciferase activities (Fluc/Rluc ×10^3^).

We used *in vitro* synthesized dicistronic viral transcripts that contained two consecutive reporters Rluc and Fluc, and monitored their expressions in RRL and compared the results obtained with those from the *Giardia* trophozoites transfected with the same transcripts [44] (Fig. 2). For the control transcript pC631Rluc-Fluc (with Rluc and Fluc separated by 10 nts), a significant Rluc activity of 5,018,431.5±284,394.9 RLU, and an approximately 100-fold lower Fluc activity of 52,848.2±7,173.2 RLU was observed, resulting in a Fluc/Rluc ratio of 10.3±0.9×10^−3^ (Fig. 2A). When the GLV 5′UTR sequence was inserted between the two cistrons of the control transcript, the F/R ratio became 5.3±1.2×10^−3^ (pC631Rluc-UTR-Fluc, Fig. 2D), whereas an insertion of the 264 nt downstream coding region from the GLV transcript (pC631Rluc-Cod-Fluc) resulted in a ratio of 9.9±0.2×10^−3^ (Fig. 2E), suggesting that neither the 5′UTR nor the 264 nts of coding region alone has the IRES activity. However, when the entire GLV IRES was placed in the inter-cistronic region (pC631Rluc-UTRCod-Fluc), the F/R ratio was 28.0±0.5×10^−3^, representing a 2 to 3-fold increase from the control value (Fig. 2F). These results indicate that, as in *Giardia*, the combination of the 5′UTR with 264 nts of capsid coding region are required for the relatively low IRES activity in RRL. When EMCV IRES or CrPV IRES was placed in between the two cistrons, the F/R ratios in RRL were raised to 591.1±25.1×10^−3^ and 89.9±5.3×10^−3^, respectively (Fig. 2, B and C), indicating that they function much more efficiently than GLV IRES. In contrast, both EMCV IRES and CrPV IRES were unable to drive Fluc expression from the dicisronic transcripts in transfected *Giardia* cells (Fig. 2, B and C), suggesting that these IRESs are inefficient in recruiting the translation machinery of *Giardia*.

### A structure-function analysis of GLV IRES in RRL

**Figure 3:**
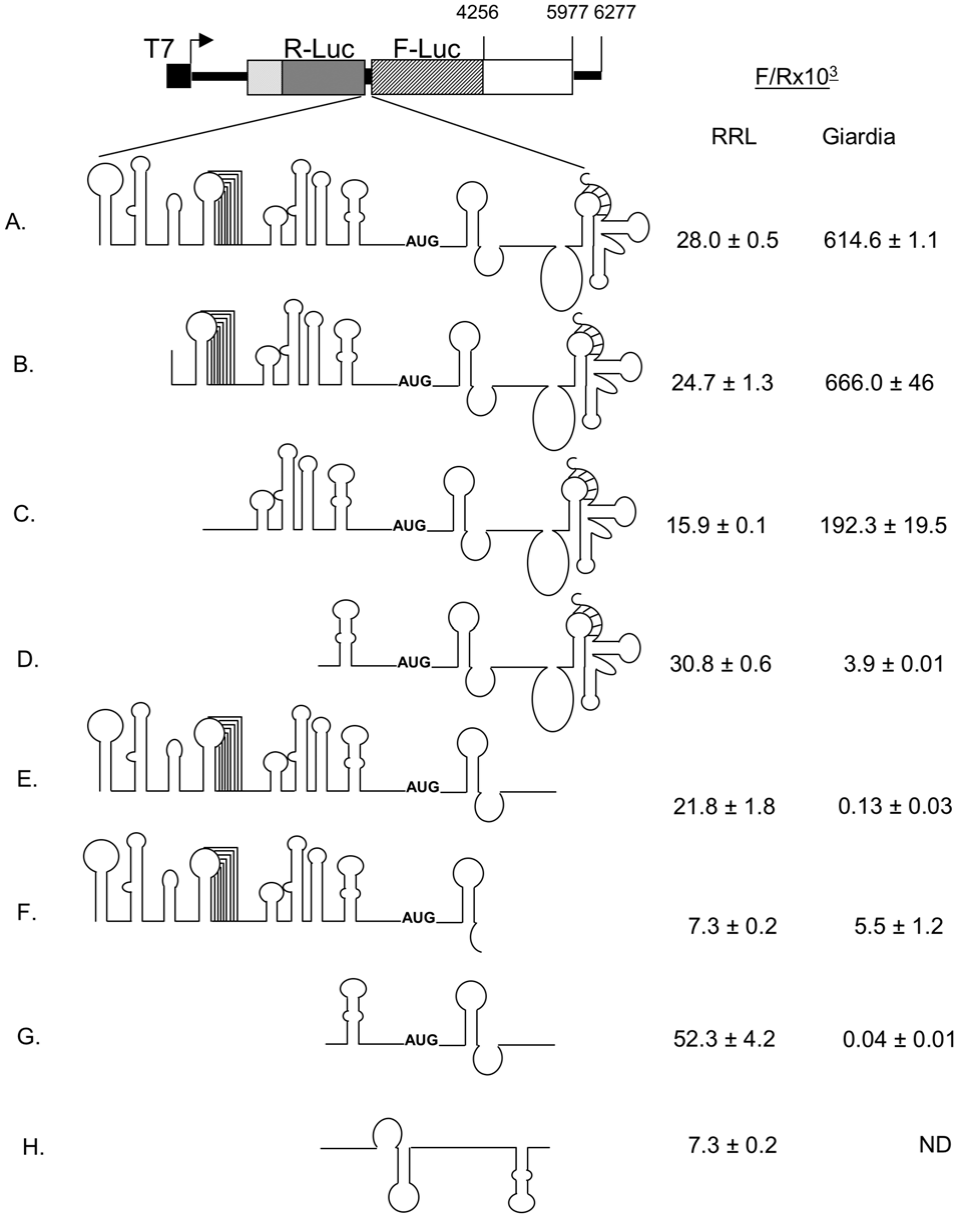
Structural requirements for GLV IRES activity in RRL and in *Giardia*. Effect of varying deletions in the 5′UTR and in the 264 nt coding region on GLV-IRES activity observed in RRL and in transfected *G. lamblia* WB trophozoites. The extent of deletion in each region is mentioned in the text and referred by the number of the transcript in the figure. The Rluc and Fluc activities were assayed from RRL reaction mixture and *Giardia* lysates. ND, not determined.

In *Giardia*, the functional GLV-IRES spans from nucleotide #114 to #631 in the viral transcript [44]. It includes essential structures such as a pseudoknot U3 (nt #134–176), stem-loops U4a (nt #204–219), U4b (nt #221–261), U4c (nt #263–292) and U5 (nt #314–344) in the 5′UTR, and stem-loop I (nt #378–402), a downstream box (Dbox) sequence (nt #433–445), and another pseudoknot (nts 511–587) in the coding region (Fig. 1). To determine if these secondary structures are also required for the limited GLV-IRES function in RRL, various deletion mutants of GLV-IRES were tested [45], [47]. The results indicated that the U3 psuedonot, U4a, b and c stem-loops from the 5′UTR (Fig. 3B–D) and the pseudoknot from the downstream capsid coding region (Fig. 3E) are not required for IRES function. The two stem-loop structures, U5 from the 5′UTR and I from the coding region, flanking a 31 nt un-structured sequence with the initiation codon AUG localized at the center was sufficient to function as an IRES in RRL. These structures were originally postulated to accommodate the recruited 40S ribosomal subunit in *Giardia* [44] and may still serve the same function in RRL. Interestingly, the results also indicated that the Dbox is apparently involved in the IRES activity in RRL (Fig. 3, compare E and F). Since Dbox was postulated to bind the 3′-end of the 16S-like ribosomal RNA in *Giardia* [50], it is not immediately clear what function it may perform in RRL. This significantly shortened GLV IRES in RRL is only 210 nts and is approximately twice as active as the full length GLV IRES in RRL (Fig. 3, G). To rule out the possibility that this enhanced activity is due to read-through of the first cistron, it was inserted into the dicistronic transcript in a inverted orientation (Fig. 3, H). The Fluc activity was completely lost, suggesting a real, albeit limited, function of this truncated GLV IRES in RRL.

### GLV IRES does not require binding of the pre-initiation complex to initiate translation

**Figure 4:**
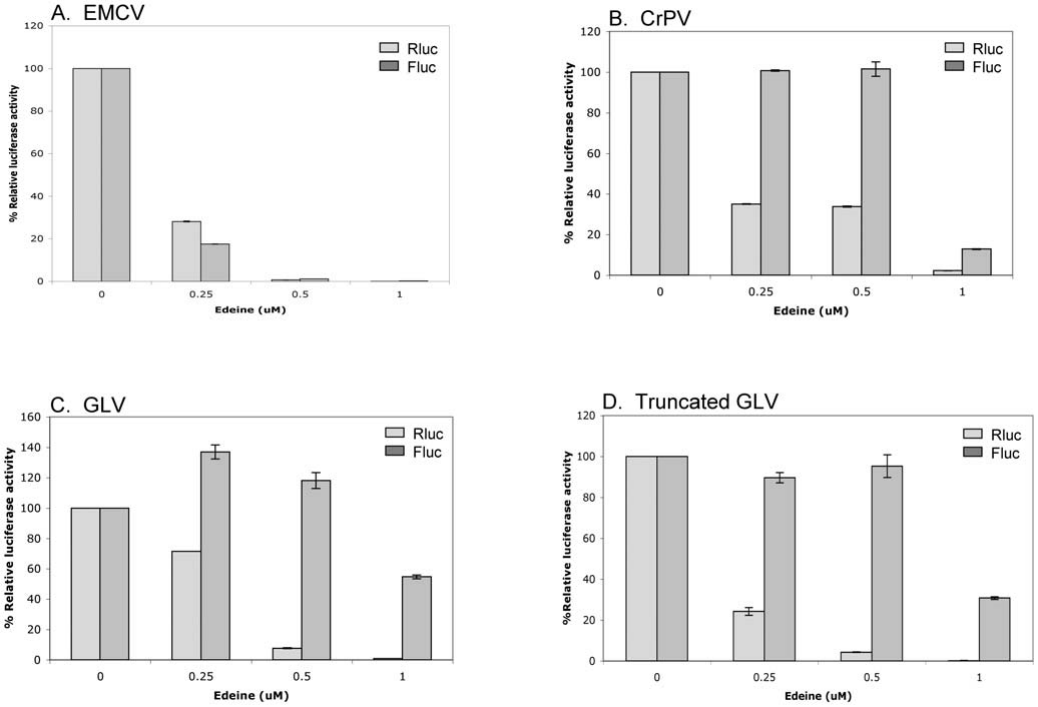
Effect of edeine on IRES functions in RRL. The translation of dicistronic transcripts (A) Cap-Rluc-EMCV IRES-Fluc, (B) Cap-Rluc-CrPV IRES-Fluc, (C) Cap-Rluc-GLV IRES (1–631 nts) Fluc and (D) Cap-Rluc-GLV IRES (276–487) Fluc in RRL, in the presence of varying concentrations of edeine was assayed. The luciferase activity of Rluc (dotted bars) and Fluc (gray bars) are presented as percentage (%) relative luciferase activities as compared to the untreated control samples (without edeine). Error bars represent standard errors from assaying triplicate samples in the same experiment.

A pre-initiation complex consisting of 40S ribosome complexed with eIF3, eIF2-GTP-Met.tRNA_i_, is required for cap mediated as well as EMCV IRES mediated translation initiation [9], [12], [13]. In contrast, CrPV IRES [51] and HCV IRES [52] do not require this pre-initiation complex as they can directly bind the 40S ribosome to initiate translation [16], [18]. These two modes of ribosome recruitment are distinguishable by using the translation initiation inhibitor edeine that prevents the recognition of the initiation codon by a pre-initiation complex in RRL [53], [54], thereby inhibits the cap-mediated and EMCV IRES driven but not the CrPV IRES and HCV IRES mediated translation initiation [51], [52], [55]. To determine if GLV IRES requires a pre-initiation complex for translation initiation, we tested 5′ capped dicistronic constructs in RRL in the presence of edeine and we found that the GLV IRES mediated translation was unaffected up to 0.5 µM of the drug (Fig. 4C), whereas the cap-mediated as well as the EMCV IRES mediated translation was inhibited by edeine in a dose dependent manner (Fig. 4A). As expected, the CrPV IRES mediated translation was also unaffected by edeine up to 0.5 µM (Fig. 4B). Interestingly, the truncated GLV IRES (nt #276–487) was also found to be insensitive to the drug up to 1 µM (Fig. 4D).

**Figure 5:**
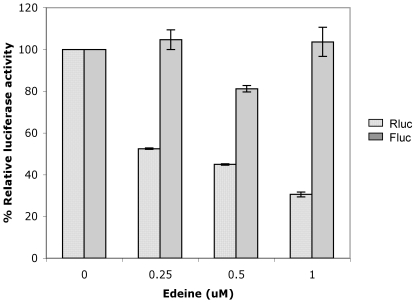
Effect of edeine on the GLV IRES activity in transfected *G. lamblia* WB trophozoites. The dicistronic transcript Cap-Rluc-GLV IRES (1–631 nt)-Fluc was electroporated into *Giardia* cells in combination with varying concentrations of edeine. The Rluc (dotted bars) and Fluc (gray bars) activities were assayed after 5 hours post transfection and expressed as relative luciferase activities compared to the no-drug control. The error bars represent the standard errors from assaying triplicate samples in the same experiment.

To determine if GLV IRES is also resistant to edeine while functioning in the transfected *Giardia*, the 5′ capped dicistronic transcript in combination with varying concentrations of edeine were co-electroporated into *Giardia* cells. Cells lysed 5 hr post-transfection were assayed for Rluc and Fluc activities. As in RRL, cap-mediated translation of the Rluc was significantly inhibited by edeine at concentrations above 0.25 µM, with a 30% inhibition achieved at 1 µM (Fig. 5). In contrast, GLV IRES activity was not inhibited even up to 1 µM of edeine (Fig. 5). These results indicate that GLV IRES does not require formation of a pre-initiation complex for initiating translation both in *Giardia* and in RRL.

### GLV-IRES does not bind to the small ribosomal subunit purified from *Giardia*

**Figure 6:**
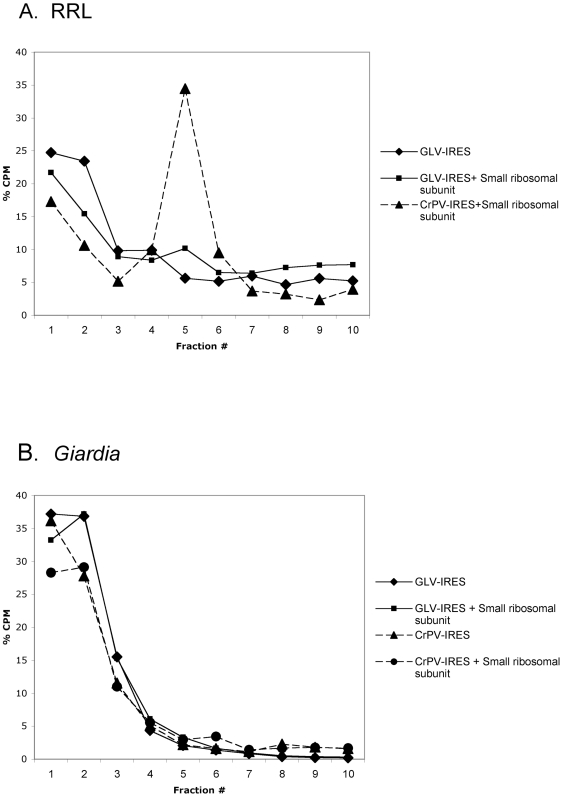
Binding of small ribosomal subunits to IRESs. Small ribosomal subunits were purified from (A) RRL and (B) *Giardia* trophozoites. They were incubated with radiolabeled GLV IRES and CrPV IRES respectively and fractionated in 10–30% sucrose density gradient centrifugations. Fractions were collected from each gradient and percent radioactivity in each fraction was recorded.

Since the function of GLV IRES was resistant to edeine, we tested its possible binding to the purified small ribosomal subunit in the absence of any initiation factors as CrPV IRES or HCV IRES are known to do with the mammalian small ribosomal subunit [16], [18]. Purified 40S ribosomal subunit from RRL was incubated with radiolabeled GLV IRES RNA or CrPV IRES RNA and the 40S-IRES RNA complexes were separated in a 10–30% sucrose density gradient. Only 10% of the labeled GLV IRES RNA was detected in the fractions containing the small ribosome subunit, indicating limited binding of GLV IRES RNA to the rabbit small ribosomal subunit. In contrast, more than 50% of the labeled CrPV IRES RNA was bound to the small subunit (Fig. 6A). To investigate if the small ribosomal subunit from *Giardia* binds to CrPV or GLV IRES RNA, we tested the latter on the small ribosomal subunit purified from *Giardia* by the same procedure as that from RRL, but no apparent binding of either IRES was detectable in the sucrose gradient (Fig. 6B) or in the filter binding assay (data not shown). The failure of CrPV-IRES in binding to *Giardia* small ribosomal subunit agrees with its failure in initiating translation in *Giardia* (see Figure 2). The lack of binding between GLV IRES and purified small ribosomal subunit from *Giardia* suggests that protein factors other than those required for pre-initiation complex formation could be required prior to the ribosomal recruitment by GLV IRES.

### Formation and analysis of IRES-protein complexes

**Figure 7:**
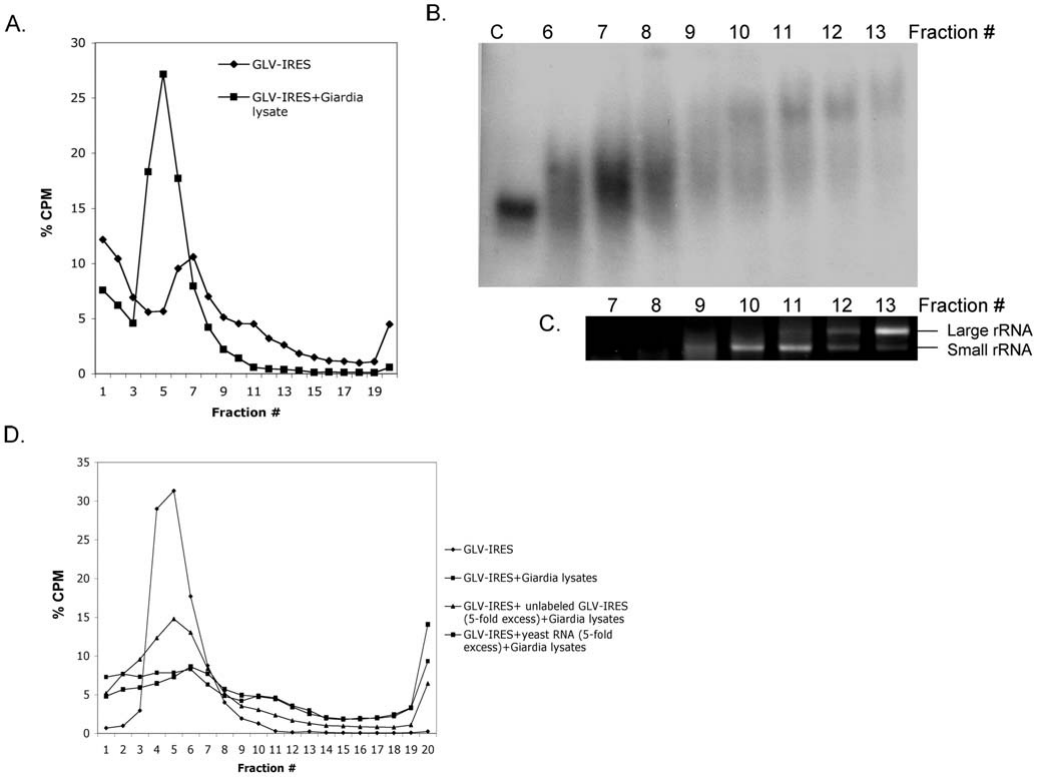
Formation of IRES-associated complexes in *Giardia* cell lysates. A. IRES-associated complexes were resolved into two peaks (Peaks 1 and 2) by 10–50% linear sucrose density gradient centrifugation. B. Analysis of sucrose density gradient fractions from #6 to #13 by composite agarose:acrylamide gel electrophoresis followed by autoradiography. C. The total RNA from fraction # 7–13 were concentrated and analyzed by agarose gel electrophoresis to visualize the presence of ribosomal RNA. D. Analysis of IRES-associated complexes formed in the *Giardia* lysate in the presence of unlabeled IRES RNA (5-fold excess) or non-specific yeast RNA (5-fold excess) (Ambion).

In order to biochemically identify the potential factors in *Giardia* that may bind to GLV IRES to enable the latter to recruit the ribosomal small subunit, we incubated the radiolabeled GLV IRES with *Giardia* cell lysates and analyzed the potential RNA-protein complexes thus formed by sucrose density gradient centrifugation. The GLV IRES-lysate mixture was separated into two distinct peaks (peaks 1 and 2) both heavier than the single GLV IRES peak in the no lysate control (Fig. 7A). The two heavier peaks were significantly reduced by a 5-fold excess of unlabeled GLV IRES but remained unchanged when an equivalent amount of random yeast RNA fragments of 300–500 nts (Ambion) was added to the mixture (Fig. 7D), suggesting that specific complexes between GLV IRES and certain components in the *Giardia* lysates were formed that constituted peaks 1 and 2. To further analyze these complexes, individual fractions collected from the gradient were each concentrated and separated on composite agarose (0.5%): acrylamide (2.75%) gels [19]. A steadily increasing shift of the radiolabeled GLV IRES RNA band toward slower mobility was clearly demonstrated from fractions #6 to #13, suggesting complexes of different sizes between GLV IRES RNA and the lysates (Fig. 7B). Peak 1 consisted of fractions #6 and #7, which had the highest radioactivity and well-defined shift in mobility, but there was no detectable ribosomal RNA in it. It may be the dominant form of GLV IRES RNA-lysate complex formed under the current experimental conditions and could be the GLV IRES RNA-ITAFs complex formed in *Giardia* prior to ribosomal recruitment. When peak 2, consisting of fractions #10 and 11, was analyzed for ribosomal RNA in an agarose gel, the 16S small ribosomal RNA band was detected primarily in fractions #10 and 11 (Fig. 7C). The peak could thus represent the complex between peak 1 and the small ribosomal subunit, i.e., the GLV IRES RNA-ITAFs-small ribosomal subunit complex, the likely initiator formed on GLV IRES.

### Affinity purification of the GLV IRES-binding proteins using StreptoTag

**Figure 8:**
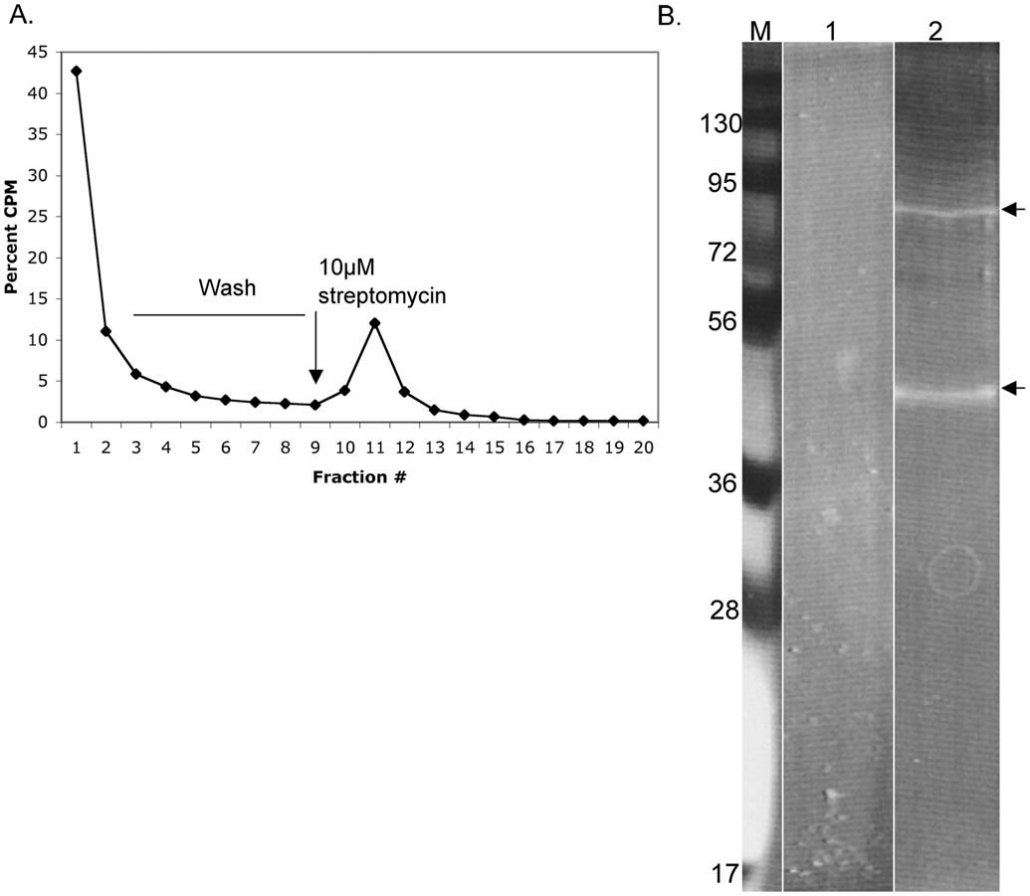
Purification of GLV IRES-associated complexes using streptomycin-sepharose 6B column chromatography. A. The elution profile of a mixture of radiolabeled Strepto-tagged IRES RNA and *Giardia* lysates through a streptomycin-sepharose 6B column. B. Syproruby stained 10% SDS-PAGE gel of the pooled fractions #10-#12 of *Giardia* lysates alone (lane 1), and the corresponding fractions of *Giardia* lysates plus the Strepto-tagged GLV-IRES RNA (lane 2).

In order to purify the GLV IRES-protein complexes formed in the *Giardia* lysate, and observed as peak 1 in sucrose density gradient (Figure 7A), we adopted a method of affinity purifying StreptoTagged RNA-protein complex through a dihydrostreptomycin coupled Sepharose 6B column [56], successfully used previously in purifying the 48S translation initiation complexes from RRL [57], [58]. Hybrid RNA containing the GLV IRES with the StreptoTag at its 3′end through a linker was synthesized. The linker contained a primer-binding site and three repeats of CU's [57] to prevent potential base pairings between GLV IRES and the StreptoTag sequence (checked by MFOLD program). The radiolabeled and StreptoTagged GLV IRES was then incubated with *Giardia* lysates and loaded onto the streptomycin-Sepharose 6B column. After several washes with the column buffer, the bound complexes were eluted with 10 µM of streptomycin. The peak fractions (#10–12, Fig. 8A) from the eluted samples were pooled, concentrated and subjected to SDS-PAGE analysis. Two major protein bands of molecular sizes ∼45 and ∼90 kDa were identified (Fig. 8B), which could be components of the peak 1 complex detected in the previous sucrose density gradient (Fig. 7B).

**Figure 9:**
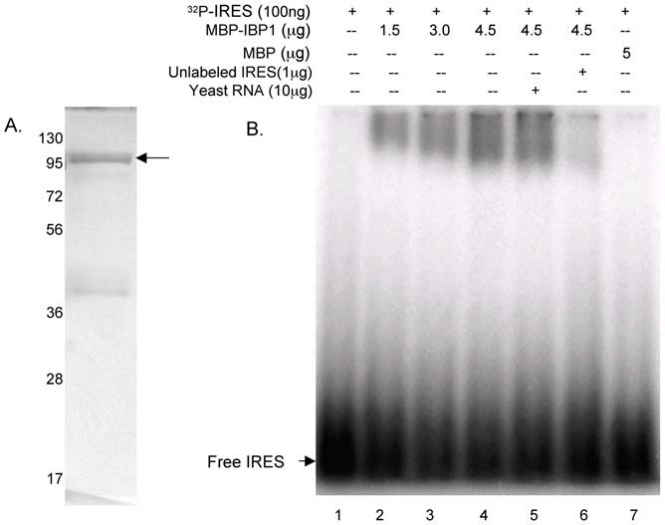
Purification of the MBP-IBP1 fusion protein expressed in *E. coli* and examination of its binding to GLV IRES RNA in gel-shift assays. A. SDS-PAGE analysis of the MBP-IBP1 fusion protein (indicated by an arrow) purified from the *E. coli* lysates using an amaylose agarose column. B. Binding of varying amounts of MBP-IBP1 to ^32^P-labeled GLV IRES RNA that was analyzed by composite agarose (0.5%):acrylamide (2.75%) gel electrophoresis and autoradiography. The RNA-protein complexes have slower gel mobility than the free RNA. Lane 1: free IRES RNA, Lanes 2–4: IRES RNA incubated with 1.5, 3.5, and 4.5 µg of MBP-IBP1 fusion protein, Lane 5, IRES RNA binding to MBP-IBP1 is unaffected by an excess of non-specific yeast RNA, Lane 6, IRES RNA binding to MBP-IBP1 is competed off by unlabeled IRES RNA, Lane 7, IRES RNA does not bind to purified MBP.

The 45 and 90 kDa protein bands were subjected to mass spectrometry using the MADLI-TOF procedure and the resulting peptides were used to identify the protein in the *Giardia* genome database. MS-FIT program matched 47 peptides in the tryptic digest of the 45 kDa band to a protein (accession # XP_770230) that belongs to the super-family I of DNA and RNA helicases, with a MOWSE score of 6.22 10*^+8^* (p<0.05) (Fig. S1). This protein hereafter is referred to as the IRES binding protein 1 (IBP1). IBP1 contains two COG1112 domains that are characteristic of Super-family I of DNA and RNA helicases, however, it does not share sequence homology with any of the RNA binding proteins nor contains conserved RNA binding motifs. The encoding DNA sequence of IBP1 was amplified by PCR, cloned, expressed as a maltose binding protein (MBP) fusion protein in transformed *Escherichia coli* and affinity purified (Fig. 9A). The purified protein was then tested for binding to GLV IRES RNA in gel-shift analysis. The mobility of radiolabeled GLV IRES was significantly reduced by increasing concentrations of MBP-IBP1 (Fig. 9B, lanes 2–4) but unaffected by MBP (Fig. 9B, lane 7). Moreover, this binding was effectively competed off by a 10-fold excess of unlabeled GLV IRES (Fig. 9B, lane 6) but not by a 100-fold excess of yeast RNA of random 300–400 nt sequences (Ambion) (Fig. 9B, lane 5). These results indicate that IBP1 specifically binds to GLV IRES. It was the first *Giardia* protein identified to bind to GLV IRES.

The peptides from the tryptic digest of the other GLV IRES-binding 90 kDa protein could not be matched to any of the existing coding sequences in the *Giardia* genome database and it was not pursued further.

### Identification in *Giardia* of homologues of trans-acting factors known to bind to other viral IRESs

**Figure 10:**
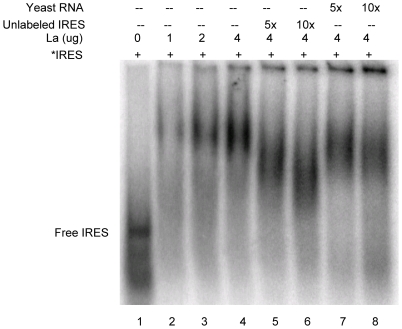
GLV IRES RNA binds to recombinant GlLa protein. Varying amounts of purified GlLa protein was incubated with radiolabeled GLV IRES RNA (lanes 2–4) and the RNA-protein complexes were separated by composite agarose (0.5%): acrylamide (2.75%) gel electrophoresis and monitored by autoradiography. The binding was reduced by 5 to 10 fold excess of unlabeled IRES RNA (lanes 5 and 6), but unaffected by 5 to 10-fold excess of non-specific yeast RNA (lanes 7 and 8).

**Figure 11:**
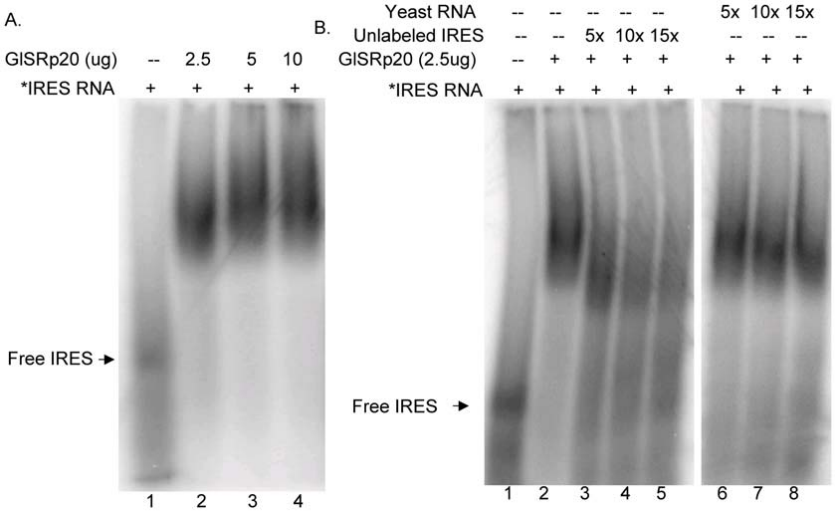
GLV IRES RNA binds to recombinant GlSRp20 protein. A. Varying amounts of purified GlSRp20 protein were incubated with radiolabeled GLV IRES RNA and analyzed by composite agaorose (0.5%): acrylamide (2.75%) gel electrophoresis and autoradiography (lanes 1–4). B. The binding was competed by 5, 10 and 15 fold-excess of unlabeled GLV IRES RNA (lanes 3–5) but not by 5, 10, and 15 fold excess of non-specific yeast RNA (Ambion) (lanes 6–8).

There have been many non-canonical *trans*-acting protein factors (ITAFs) identified in higher eukaryotes that are found to bind and stimulate the activity of various viral IRESs [3]. These ITAFs were used to search for homologues in the *Giardia* genome database (http://giardiadb.org/giardiadb/). A homologue of La autoantigen, which was found to bind HCV, EMCV, PV and Coxsackie virus IRES elements [21], [23], [26], [27], a homologue of SRp20, which is known to bind to Poliovirus IRES [32] and a homologue of hnRNP E2 involved in binding to PV IRES [34] were identified in *Giardia*. The La homologue in *Giardia* contains the characteristic La domain within the first 100 amino acids, followed by the RNA recognition motifs (RRM) 2 and 3, and a divergent C-terminal domain (Fig. S2), whereas SRp20 shares significant sequence identity with mouse and human SRp20 only at the N terminus (Fig. S3). In contrast to human hnRNPE2, *Giardia* hnRNPE2 contained only a single KH domain (data not shown). DNA fragments encoding the three homologues (GlLa, accession # XP_001705495; GlSRp20, accession # XP_001708843; GlhnRNPE2, accession # XP_00170795) were cloned, expressed in the form of 6X-His tagged fusion proteins in transformed *E. coli*, and affinity purified (Fig. S4). The purified proteins were tested for their binding to GLV IRES in the gel shift assays. GlLa showed a strong binding to GLV IRES RNA in a dose dependent manner (Fig. 10, lanes 2–4). This binding was significantly reduced in the presence of 5 to 10-fold excess of unlabeled GLV IRES (Fig. 10, lanes 5–6) but unaffected by an excessive amount of yeast RNA (Fig. 10, lanes 7–8). Similarly, GlSRp20 also exhibited binding to radiolabeled GLV IRES in a dose dependent manner (Fig. 11A, lanes 2–4), which was significantly reduced by 15-fold excess of unlabeled GLV-IRES (Fig. 11B, lanes 3–5) but unaffected by excessive yeast RNA (Ambion) (Fig. 11B, lanes 6–8) indicating specific binding of GlSRp20 to GLV IRES. In contrast to GlSRp20 and GlLa proteins, recombinant GlhnRNPE2 did not show detectable binding to radiolabeled GLV IRES RNA (data not shown). Thus, two additional proteins in *Giardia*, GlSRp20 and GlLa, demonstrating specific binding to GLV IRES were identified. Based on the known functions of their homologues in higher eukaryotes, the potential functions of GlLa and GlSRp20 on GLV IRES activity in *Giardia* could be postulated.

**Figure 12:**
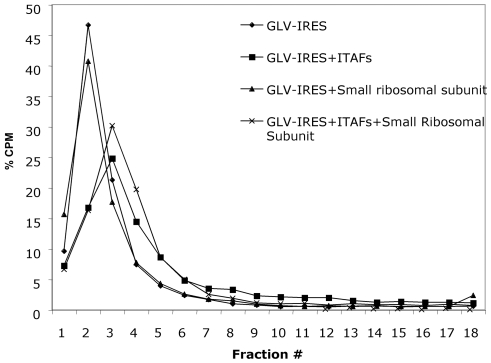
The lack of effect of binding of the three ITAFs to GLV IRES on recruiting *Giardia* small ribosomal subunit. Radiolabeled GLV IRES alone (diamond), GLV IRES incubated with the small ribosomal subunit (triangle), GLV IRES incubated with the ITAFs GlIBP1, GlLa, and GlSRp20 (square) or plus the small ribosomal subunit (star) were each fractionated in a 10–30% sucrose density gradient centrifugation. Each gradient was collected in fractions, and the radioactivity was counted.

To determine the potential roles of the three putative *trans*-acting protein factors GlIBP1, GlLa and GlSRp20 in GLV IRES mediated translation initiation, we tested whether their bindings to GLV IRES would help recruit the *Giardia* small ribosomal subunit. The result shown in Figure 12 indicates that the RNA-protein complex is incapable of recruiting the small ribosomal subunit. Apparently, the three proteins do not constitute the entire spectrum of ITAFs needed for ribosomal recruitment. But the shift of the RNA-protein complex toward heavier fractions in the sucrose density gradient suggests that additional proteins may be needed to join the complex to make the complex we observed in peak 1 in Figure 7A between GLV IRES and *Giardia* lysates.

## Discussion

Our study shows that GLV IRES is functional in RRL system despite the evolutionary divergence between *Giardia* and rabbit. However, it functions at a much lower efficiency than EMCV IRES or CrPV IRES. This GLV IRES function in RRL requires an significantly truncated structure involving only the upstream stem-loop U5 and the downstream stem-loop I plus the Dbox, flanking the AUG codon in the center. This is the structure amid a highly complex assembly of secondary structures when the original IRES was found to function in *Giardia* [44]. Requirement of additional structures for optimal IRES activity in *Giardia* could reflect the functional differences between the translation machineries of *Giardia* and RRL.

Our data also indicate that GLV IRES may follow a distinct pathway for forming an initiation complex. Resistance of GLV IRES to the translation initiation inhibitor, edeine, indicates that a 40S-eIF2-GTP/Met-tRNA_i_ pre-initiation complex is not required for initiating translation. Edeine has been shown to bind between the P- and the E-sites of the ribosome and interfere with the recognition of the AUG codon by a scanning pre-initiation complex [53], [54], [59]. The resistance of CrPV IRES to edeine inhibition is attributed to its ability to bind directly to the small ribosomal subunit and position pseudoknot structure PKI in the P-site of the ribosome, and initiate translation from the non-canonical GCU codon positioned at the ribosomal A-site [55], [60], [61]. Similarly, HCV IRES has also been shown to resist edeine inhibition at relatively low concentrations [52], [62] and yet it initiates translation from the AUG codon located at the P-site of the ribosome [17], [52], by first binding directly to the small ribosomal subunit in the absence of initiation factors and then recruiting the eIF3 and eIF2-GTP-tRNA ternary complex [16], [17], [63], [64]. Since GLV IRES does not share sequence or structural similarity with CrPV IRES or HCV IRES, the mechanism of its resistance to edeine could be quite different. GLV IRES utilizes the single AUG located between stem-loop U5 and I to initiate translation [46]. It may be employing a mechanism resembling that of the HCV IRES, involving initial binding of the small ribosomal subunit to form initiation complexes that bypasses the inhibitory effect of edeine. But the failure of GLV IRES in directly binding to *Giardia* small ribosomal subunit suggested a more complex situation. It is well established that the ability of CrPV IRES and HCV IRES to directly bind to small ribosomal subunit is due to their inherent nature to fold into a compact three-dimensional structure containing specific ribosome binding domains [16], [18], [65], [66]. The lack of GLV IRES binding to the *Giardia* small ribosomal subunit could be attributed to an inability to fold into such a compact three-dimensional structure. The secondary and tertiary structures in the 5′UTR region of GLV IRES are each separated by single stranded regions [47]. They may not form a compact high-order structure on their own but may bind to protein factors known to promote structural organization of an IRES to form a structure capable of binding directly to the small ribosomal subunit [10], [11]. The detection of peaks 1 and 2 from a GLV IRES and *Giardia* lysate mixture in a sucrose gradient is consistent with this possibility. IBP1, identified in this mixture, is most likely playing a role in the binding between the small ribosomal subunit and GLV IRES.

In addition to IBP1, we identified a homologue of La autoantigen in *Giardia* that binds to GLV IRES specifically *in vitro*. Human La autoantigen is a dimer and contains several RNA binding domains [24]. It binds to a specific region (nt position 559–624) in the PV IRES and stimulates the 48S complex formation both *in vitro* and *in vivo* [22], [24]. It also binds near the initiation codon of the HCV IRES to stimulate the IRES activity [23], presumably through stabilizing the IRES structure and thus facilitating the interaction with the translation machinery [67]. Since, La-motif containing proteins are highly conserved among eukaryotes [68], GlLa could have similar functions as human La. It could be stabilizing the structure of GLV IRES and thus facilitating the binding of small ribosomal subunit.

SRp20 has been reported to stimulate PV IRES activity by interacting with hnRNPE2 [32] and to co-sediment with the 80S ribosome and polysomes [69]. GlSRp20, showing specific binding to GLV IRES *in vitro*, could have a similar function on GLV IRES through a direct interaction with the small ribosomal subunit.

In conclusion, our data indicate that GLV IRES utilizes a distinctive pathway of recruiting the small ribosomal subunit that does not require a pre-initiation complex. Inability of the three ITAFs to stimulate recruitment of the small ribosomal subunit to GLV IRES indicates that additional protein factors are needed. Further studies to identify other components in the peaks 1 and 2 of GLV IRES and lysate mixture (Fig. 7) are required for elucidating the mechanism of GLV IRES mediated translation initiation.

## Materials and Methods

### Construction of dicistronic vectors

Construction of plasmids pC631Rluc-Fluc, pC631Rluc-UTR-Fluc, pC631Rluc-Cod-Fluc, pC631Rluc-UTRCod-Fluc, pC631Rluc-114-367Cod-Fluc, pC631Rluc-126-367Cod-Fluc, pC631Rluc-176-367Cod-Fluc and pC631Rluc-278-367Cod-Fluc has been described previously [44]. For construction of pC631Rluc-EMCV-Fluc ∼600 bp *Xho*I/*Hind*III fragment containing EMCV IRES was excised from pIRES (Clontech) and inserted into the *Xho*I/*Hind* III site upstream of the Fluc gene in pC631Rluc-Fluc. For pC631Rluc-CrPV-Fluc, CrPV IRES was amplified by PCR as a *Xho*I/*Hind*III fragment using CrPV1-1 (Eric Jan, University of British Columbia) as template and cloned into the *Xho*I/*Hind*III site of pC631Rluc-Fluc. Similar strategies were employed for construction of pC631Rluc-UTRCodΔ1-Fluc, pC631Rluc-UTRCodΔ2-Fluc and pC631Rluc-278CodΔ2-Fluc. The UTRCodΔ1, UTRCodΔ2 and 278-CodΔ2 sequences were amplified as *Xho*I/*Hind*III fragments and cloned into pC631Rluc-Fluc. To generate pC631Rluc-278CodΔ2rev-Fluc, a 278-CodΔ2 sequence was amplified as *Hind*III/*Xho*I fragment and was ligated with *Xho*I/*Hind*III digested pC631Rluc-Fluc.

For generation of capped transcripts, Renilla luciferase gene (*Rluc*) was PCR amplified using a pNull-Rluc plasmid (Promega) as template and the product was inserted downstream of T7 promoter using the *Nhe*I and *Xho*I sites in pIRES vector (Clontech) to generate pIRES-Rluc. A UTRCod-Fluc region was PCR amplified from pC631Rluc-UTRCod-Fluc as a *Xho*I/*Xba*I fragment, cloned into pGEM-T vector (Promega), excised as a *Xho*I/*Not*I fragment and inserted into the *Xho*I/*Not*I site located downstream of the Rluc gene in pIRES-Rluc. To generate transcripts containing a 50 polyA tail, annealed oligonucleotides 5′ CTAGA_51_G 3′ and 5′ AATTCT_51_ 3′ were inserted into the *Xba*/*Eco*RI site downstream of the Fluc gene in the pIRES-Rluc-UTRCod-Fluc plasmid.

### In vitro transcription

The dicistronic constructs in pC631 plasmid were linearized using *Nru* I and used as templates for *in vitro* synthesis of transcripts using MegaScript T7 transcription kit (ambion). For synthesis of capped and polyadenylated transcripts, the dicistronic constructs in pIRES plasmids were linearized with *Eco*RI and used as templates in the mMessage mMachine T7 transcription kit (Ambion).

### In vitro translation assays

Dicistronic transcripts were expressed in rabbit reticulocyte lysate (RRL) by using Flexi-Rabbit reticulocyte lysate system according to the manufacturer's instructions (Promega). Briefly, 0.5–1.0 µg of the transcript was added to a 25 µL reaction mixture and was incubated at 37° C for 90 min. Equal amounts of transcripts were used for each experiment. Reaction products (2.5 µL) were assayed for Fluc and Rluc activities using Dual-luciferase reporter assay system (Promega). To test the effect of edeine on translation, different concentrations of the drug were added to the reaction mixture and incubated on ice for 5 minutes prior to the addition of the transcript.

### Transfection of *Giardia* trophozoites and the luciferase assay

Transient transfection of *Giardia* trophozoitess was carried out as previously described [43], [45]. The cells were harvested after 5 or 16 hours of post-transfection and the cell lysate was assayed for Fluc and Rluc activities by using a Dual luciferase assay system (Promega) [44]. To test the effect of edeine on IRES activity, the drug was mixed with the *in vitro* synthesized transcripts and electroporated into *Giardia* cells. The concentrations of drug indicated in Figure 6, represent its concentration in the electropration mix of 400 µL.

### Purification of 40S ribosomal subunits and sucrose gradient centrifugation

The 40S ribosomal subunits from RRL were prepared as described [16]. Polysomes from *Giardia* trophozoites were prepared [70] by gently lysing the cell by dounce homogenizer and pelleted at 78,000xg for 4 hours in a TLA 100.3 rotor (Beckman), and resuspended in buffer A (20 mM Tris-Cl, pH 7.5, 4 mM MgCl2, 50 mM KCl, 2 mM DTT, complete protease inhibitor) to a final concetration of 100 OD_260_/mL. Puromycin A was added to 1 mM and incubated on ice for 10 min followed by another 10 min at 37 C. To the treated polysomes, 2.5 M KCl was slowly added to 0.5 M and layered on to 10–30% sucrose gradient in buffer B (20 mM Tris-Hcl, pH 7.5, 0.5 M KCl, 3 mM MgCl2, 2 mM DTT) and centrifuged at 64,000xg in a SW 55 rotor at 4°C for 17 hrs. Fractions (0.2 mL) were collected and checked for rRNA by agarose gel electrophoresis. The fractions containing separated 40S and 60S subunits were pooled and concentrated using a centricon-30 concentrator and exchanged with buffer C (0.24 M sucrose, 20 mM Tris, pH 7.5, 10 mM KCl, 1 mM MgCl2, 1 mM DTT, 0.1 mM EDTA).

The purified mammalian 40S subunits or *Giardia* small ribosomal subunits (150 nM) were incubated with 5′end labeled GLV IRES RNA (10–60 ng) in buffer E (20 mM Tris-Hcl, pH 7.5, 100 mM KOAc, 2.5 mM MgOAc, 0.25 mM spermidine, 2 mM DTT) and incubated at room temperature for 30 min. The IRES-ribosomal complexes were then layered onto 10–30% linear sucrose gradient in buffer E and centrifuged at 200, 000 x g in a SW41 rotor at 4°C for 2 hours and 10 min. The gradient was fractionated (500 uL each) and the radioactivity was counted. As control, labeled IRES RNA was incubated under similar conditions in the absence of mammalian 40S ribosomal subunits or Giardia small ribosomal subunit and was centrifuged through the gradient along with the test samples. To test the role of trans-acting protein factors, 4 µg each of GlIBP1, GlLa and GlSRp20 were pre-incubated with radiolabeled GLV IRES for 5 min at room temperature and then further incubated with purified *Giardia* small ribosomal subunit for additional 15 minutes. The reaction mixtures were then separated on a 10–30% sucrose gradient.

### Preparation of cell extracts and analysis of IRES-protein complexes

*Giardia* cell lysate was prepared as described by Bergamini et al., [71]. Briefly, 200 mL of logarithmic *Giardia* trophozoites were harvested by centrifugation at 5,000xg at 4°C for 10 min and washed three times with phosphate buffered saline (PBS) and suspended in 200 µL of lysis buffer (10 mM HEPES, pH 7.6, 10 mM KOAc, 0.5 mM MgOAc, 5 mM DTT, complete protease inhibitors minus EDTA). The cells were lysed by gentle sonication on ice for 5 minutes with 10-second pulses at 20-second intervals. The cell lysate was centrifuged at 10,000xg for 10 min. at 4°C and the cleared supernatant was used for the complex formation. The 10 µL (1.25 OD at 260 nm) cleared supernatant was pre-incubated with 0.5 U/µL of RNasin (Promega) at 30°C for 10 minutes to inhibit ribonucleases and then incubated with 3 µg of uniformly radiolabeled GLV IRES RNA (631 nts) in incubation buffer (16 mM HEPES, pH 7.6, 50 mM KOAc, 2.5 mM MgOAc, 0.1 mM spermidine, 1 mM DTT) [71] at 30°C for an additional 20 min in a final volume of 50 µL. The reaction mixture was layered on top of a 10 mL 10–40% linear sucrose density gradient (in reaction buffer) and centrifuged at 78,000xg at 4°C in a SW41 rotor (Beckman) for 16 hours. The gradients were fractionated and the radioactivity in each fraction was counted using liquid scintillation counting.

### Purification of the IRES-associated complexes by the StreptoTag method

Two complementary primers containing the aptamer sequence [56], [57] were annealed and inserted into Hind III/EcoRI sites located at the 3′ end of the IRES sequence in pC631 plasmid. The recombinant plasmid linearized by EcoRI was used as a template for *in vitro* transcription reactions. The tagged IRES RNA molecules were made using T7 megascript transcription kit (Ambion) in the presence of trace amounts of ^32^P UTP. The radiolabeled RNA was purified using RNeasy purification kit (Qiagen) and the purified RNA was suspended in folding buffer (Tris-HCl, pH 7.5, NaCl 150 mM, 3 mM MgCl_2_). To allow the proper folding of the IRES and the streptoTag aptamer, the hybrid RNA molecules were heated at 65°C for 5 min, followed by 10 min at 37° C and then cooled to room temperature.

The IRES-protein complexes were loaded onto the dihydrostreptomycin coupled Sepharose 6B coulumn (1 mL bead volume) and washed with 10 column volumes of wash buffer (16 mM Hepes, pH 7.4, 50 mM KOAc, 2.5 mM MgCl_2_, 6.8% sucrose) at room temperature [56]. The complexes were then eluted with five column volumes of wash buffer containing 10 µM streptomycin. Since hybrid RNA molecules were radiolabeled, the entire purification process was monitored using a radioactive counter. The streptomycin-eluted fractions (1 mL each) with high radioactive counts were pooled and the purified complexes were pelleted at 100,000xg in a TLA100.3 rotor at 4°C for 12–16 hours. The pellets were suspended in wash buffer and analyzed by SDS-PAGE. The protein bands were stained with Sypro Ruby (Invitrogen) and visualized by UV-light.

### Protein identification by Mass Spectrometry (MALDI-TOF)

The Sypro Ruby stained protein bands were excised from the SDS-PAGE gels and subjected to in-gel trypsin digestion [49]. The digested peptides were cleaned with ZipTipC18 and analyzed with a matrix-assisted laser desorption ionization-time of flight (MALDI-TOF) mass spectrometry instrument (Voyager DE-STR mass spectrometer, Applied Biosystems). The resulting peptide masses were used to query the *Giardia* genome database using MS-FIT of the ProteinProspector program (http://prospector.ucsf.edu/prospector/4.0.7/htm/msfit/htm).

### Cloning and purification of IRES binding proteins

The coding sequence for the IBP1 protein was amplified and cloned into expression vector pMAL-C2X (New England Biolabs) and was expressed as Maltose binding protein (MBP) fusion protein in *E. coli* cells. The expressed MBP-IBP1 fusion protein was purified by amylose-agarose chromatography following the manufacturer's instructions (New England Biolabs). The GlSRp20 and GlLa coding sequences were cloned into expression vector pET28b (Qiagen), and expressed as 6XHis tagged proteins in *E. coli*. The expressed proteins were purified from the cell lysates using Ni-NTA agarose affinity chromatography (Qiagen).

### Gel Shift assays

Approximately 100 ngs of 5′ radiolabeled IRES RNA was mixed with varying amounts of purified protein in the binding buffer (20 mM Tris, pH 7.6, 50 mM KCl, 2.5 mM MgOAc, 0.05% NP40, 1 mM DTT and 1 µg/µL of yeast tRNA) and incubated at 30°C for 20 min. The IRES-protein complexes were separated in a composite agarose (0.5%): acrylamide (2.75%) gel (Jan, Kinzy and Sarnow, 2003) and visualized by phosphorimager (Amersham).

## Supporting Information

Figure S1 The amino acid sequence of IBP1 identified by Mass spectrometry. The underlined sequences represent the peptides that were identified in mass spectrometry and used to identify the protein in the Giardia genome database.
(0.09 MB TIF)

Figure S2 Amino acid sequence alignment of Giardia La protein with homologues from Trypanosoma brucei, Drosophila melanogaster, human and yeast. The La motif, RRM2 and RRM3 domains are marked with colored lines.
(0.88 MB PDF)

Figure S3 Amino acid sequence alignment of Giardia SRp20 protein with homologues from human and mouse. The RRM motif is indicated by colored line.
(0.26 MB PDF)

Figure S4 SDS-PAGE analysis of the purified 6xHis tagged GiLa (lane 2) and GlSRp20 (lane 3) from E.coli.
(0.07 MB PDF)
