# Supplementary figures and images for: Giardiavirus Internal Ribosome Entry Site Has an Apparently Unique Mechanism of Initiating Translation
Source: PLoS One. 2009 Oct 14;4(10):e7435. doi: 10.1371/journal.pone.0007435 (PMC2757703; doi:10.1371/journal.pone.0007435)

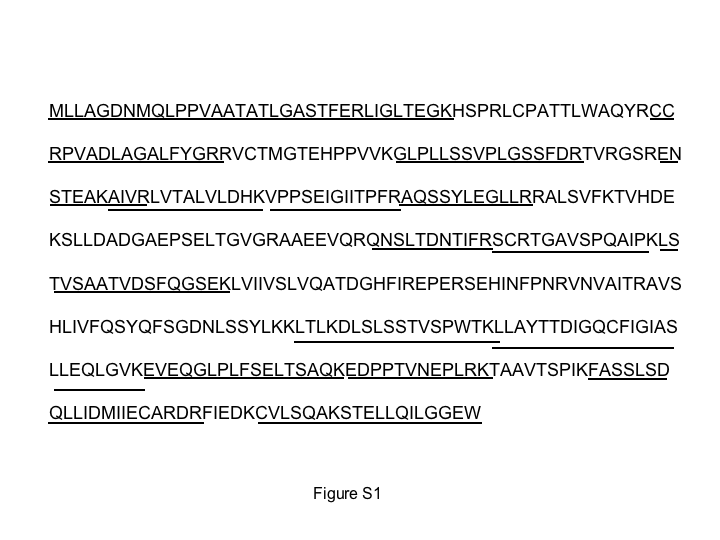

Supplement: Figure S1 — The amino acid sequence of IBP1 identified by Mass spectrometry. The underlined sequences represent the peptides that were identified in mass spectrometry and used to identify the protein in the Giardia genome database. (0.09 MB TIF) [file pone.0007435.s001.tif]

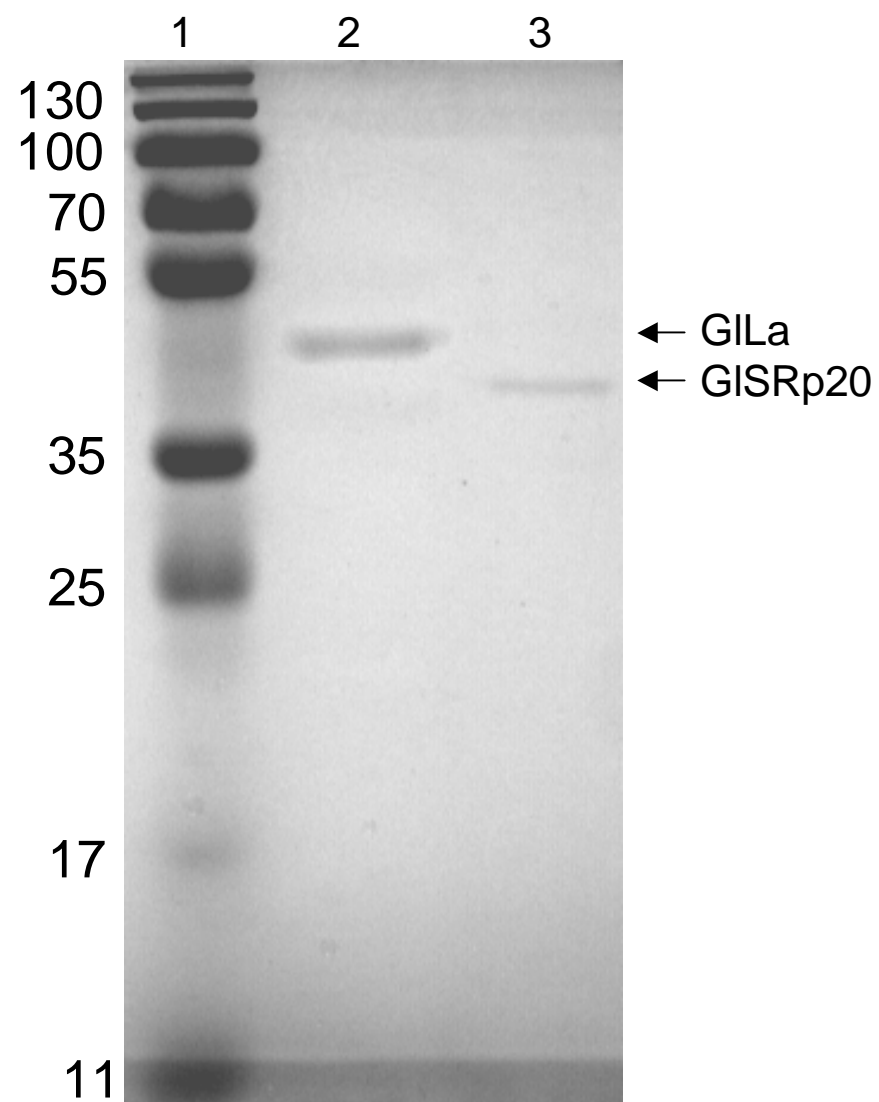

Figure S4

Supplement: Figure S4 — SDS-PAGE analysis of the purified 6xHis tagged GiLa (lane 2) and GlSRp20 (lane 3) from E.coli. (0.07 MB PDF) [file pone.0007435.s004.pdf]
