# Supplementary material for: Giardiavirus Internal Ribosome Entry Site Has an Apparently Unique Mechanism of Initiating Translation
Source: PLoS One. 2009 Oct 14;4(10):e7435. doi: 10.1371/journal.pone.0007435 (PMC2757703; doi:10.1371/journal.pone.0007435)
Supplement: Figure S2 — Amino acid sequence alignment of Giardia La protein with homologues from Trypanosoma brucei, Drosophila melanogaster, human and yeast. The La motif, RRM2 and RRM3 domains are marked with colored lines. (0.88 MB PDF) [file pone.0007435.s002.pdf]

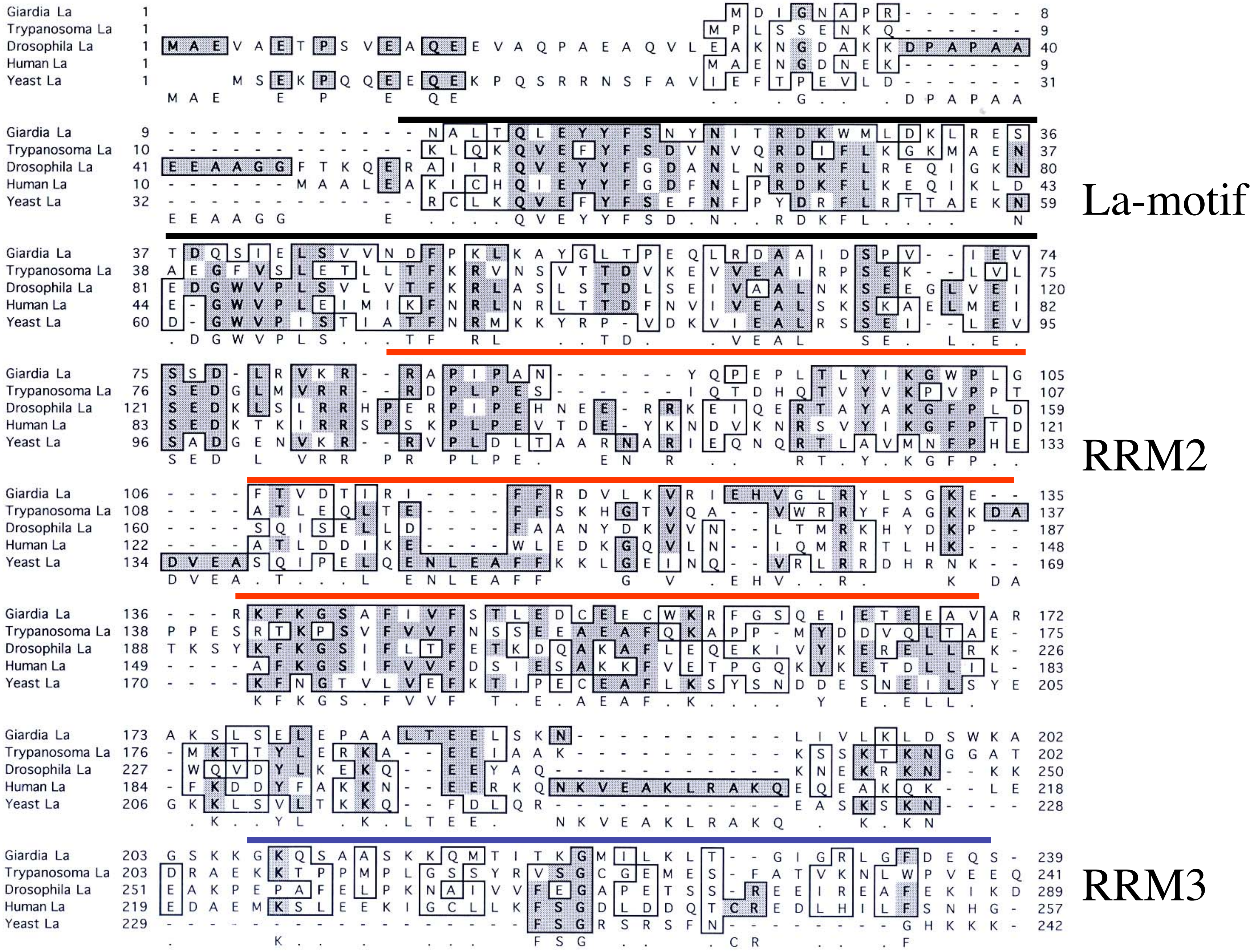
Giardia La
Trypanosoma La
Drosophila La
Human La
Yeast La
La-motif
RRM2
RRM3

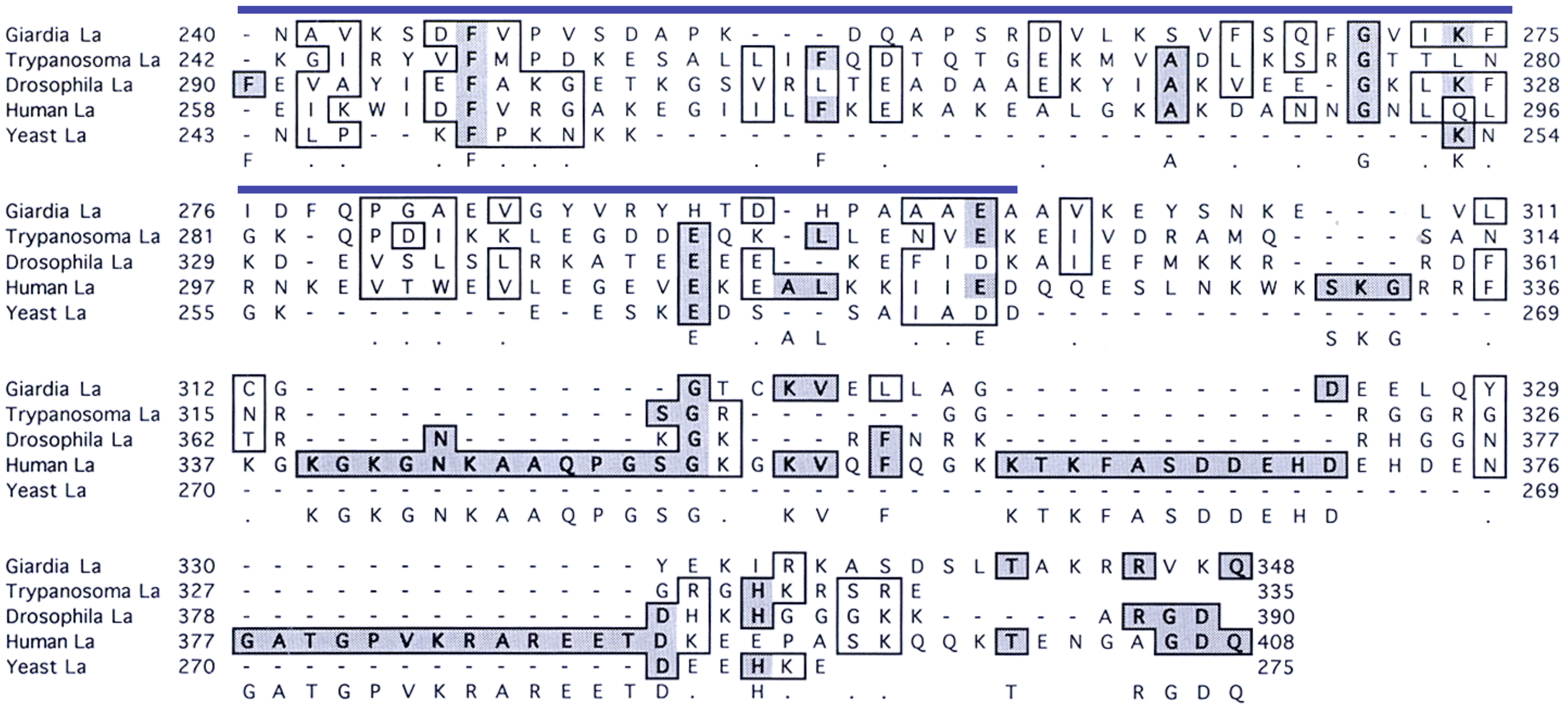

Figure S2
