# Supplementary material for: Giardiavirus Internal Ribosome Entry Site Has an Apparently Unique Mechanism of Initiating Translation
Source: PLoS One. 2009 Oct 14;4(10):e7435. doi: 10.1371/journal.pone.0007435 (PMC2757703; doi:10.1371/journal.pone.0007435)
Supplement: Figure S3 — Amino acid sequence alignment of Giardia SRp20 protein with homologues from human and mouse. The RRM motif is indicated by colored line. (0.26 MB PDF) [file pone.0007435.s003.pdf]

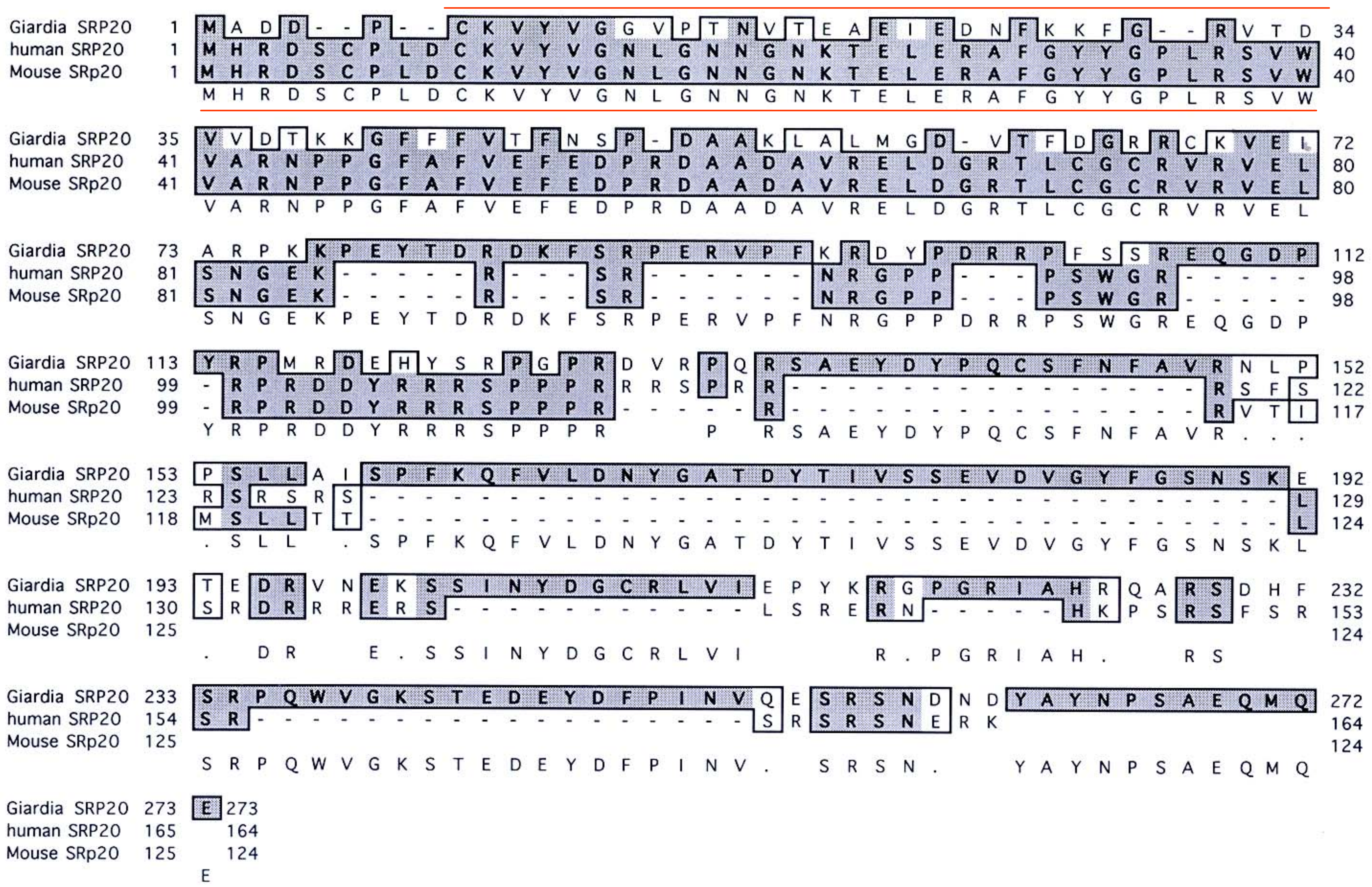
Giardia SRP20 1 M A D D - - P - - C K V Y V G G V P T N V T E A E I E D N F K K F G - - R V T D 34
human SRP20 1 M H R D S C P L D C K V Y V G N L G N N G N K T E L E R A F G Y Y G P L R S V W 40
Mouse SRp20 1 M H R D S C P L D C K V Y V G N L G N N G N K T E L E R A F G Y Y G P L R S V W 40
M H R D S C P L D C K V Y V G N L G N N G N K T E L E R A F G Y Y G P L R S V W
Giardia SRP20 35 V V D T K K G F F F V T F N S P - D A A K L A L M G D - V T F D G R R C K V E L 72
human SRP20 41 V A R N P P G F A F V E F E D P R D A A D A V R E L D G R T L C G C R V R V E L 80
Mouse SRp20 41 V A R N P P G F A F V E F E D P R D A A D A V R E L D G R T L C G C R V R V E L 80
V A R N P P G F A F V E F E D P R D A A D A V R E L D G R T L C G C R V R V E L
Giardia SRP20 73 A R P K K P E Y T D R D K F S R P E R V P F K R D Y P D R R P F S S R E Q G D P 112
human SRP20 81 S N G E K - - - - - - R - - - S R - - - - - - N R G P P - - - P S W G R - - - - - 98
Mouse SRp20 81 S N G E K - - - - - - R - - - S R - - - - - - N R G P P - - - P S W G R - - - - - 98
S N G E K P E Y T D R D K F S R P E R V P F N R G P P D R R P S W G R E Q G D P
Giardia SRP20 113 Y R P M R D E H Y S R P G P R D V R P Q R S A E Y D Y P Q C S F N F A V R N L P 152
human SRP20 99 - R P R D D Y R R R S P P P R R R S P R R - - - - - - - - - - - - - - - R S F S 122
Mouse SRp20 99 - R P R D D Y R R R S P P P R - - - - - R - - - - - - - - - - - - - - - R V T I 117
Y R P R D D Y R R R S P P P R P R S A E Y D Y P Q C S F N F A V R . . .
Giardia SRP20 153 P S L L A I S P F K Q F V L D N Y G A T D Y T I V S S E V D V G Y F G S N S K E 192
human SRP20 123 R S R S R S - L 129
Mouse SRp20 118 M S L L T T - L 124
. S L L . S P F K Q F V L D N Y G A T D Y T I V S S E V D V G Y F G S N S K L
Giardia SRP20 193 T E D R V N E K S S I N Y D G C R L V I E P Y K R G P G R I A H R Q A R S D H F 232
human SRP20 130 S R D R R R E R S - - - - - - - - - - - L S R E R N - - - - - - H K P S R S F S R 153
Mouse SRp20 125 124
. D R E . S S I N Y D G C R L V I R . P G R I A H . R S
Giardia SRP20 233 S R P Q W V G K S T E D E Y D F P I N V Q E S R S N D N D Y A Y N P S A E Q M Q 272
human SRP20 154 S R - - - - - - - - - - - - - - - - - - S R S R S N E R K 164
Mouse SRp20 125 124
S R P Q W V G K S T E D E Y D F P I N V . S R S N . Y A Y N P S A E Q M Q
Giardia SRP20 273 E 273
human SRP20 165 164
Mouse SRp20 125 124
E

Figure S3
